# Plasticity and genetic adaptation mediate amphibian and reptile responses to climate change

**DOI:** 10.1111/eva.12114

**Published:** 2013-10-30

**Authors:** Mark C Urban, Jonathan L Richardson, Nicole A Freidenfelds

**Affiliations:** Department of Ecology and Evolutionary Biology, University of ConnecticutStorrs, CT, USA

**Keywords:** common garden experiments, contemporary evolution, global change, local adaptation, phenotypic plasticity

## Abstract

Phenotypic plasticity and genetic adaptation are predicted to mitigate some of the negative biotic consequences of climate change. Here, we evaluate evidence for plastic and evolutionary responses to climate variation in amphibians and reptiles via a literature review and meta-analysis. We included studies that either document phenotypic changes through time or space. Plasticity had a clear and ubiquitous role in promoting phenotypic changes in response to climate variation. For adaptive evolution, we found no direct evidence for evolution of amphibians or reptiles in response to climate change over time. However, we found many studies that documented adaptive responses to climate along spatial gradients. Plasticity provided a mixture of adaptive and maladaptive responses to climate change, highlighting that plasticity frequently, but not always, could ameliorate climate change. Based on our review, we advocate for more experiments that survey genetic changes through time in response to climate change. Overall, plastic and genetic variation in amphibians and reptiles could buffer some of the formidable threats from climate change, but large uncertainties remain owing to limited data.

## Introduction

Climate change is forcing species to pursue their climatic niche as it shifts across landscapes (Chen et al. 2011). Some species will not track their optimal climates because they disperse poorly or they cannot disperse across natural or human barriers (Loarie et al. 2009; Schloss et al. 2012; Urban et al. 2013). These species face the threat of high extinction rates (Thomas et al. 2004; Malcolm et al. 2006; Schloss et al. 2012). However, phenotypic plasticity and genetic adaptation could allow these species to persist in their current locations despite climate change. Here, we review studies that examine evidence for plastic or genetic changes in amphibians and reptiles in response to climate variation.

Amphibians and reptiles might be particularly sensitive to climate change. Both taxa have already experienced extensive declines and extinctions worldwide (Gibbons et al. 2000; Houlahan et al. 2000; Blaustein and Kiesecker 2002; Sinervo et al. 2010). Both groups have been called ‘canaries in the coal mine’ based on their supposed sensitivity to environmental change (Kerby et al. 2010; Mitchell and Janzen 2010). A global assessment further suggests that amphibians are declining faster than birds or mammals (Stuart et al. 2004). Concurrently, Huey et al. (2010) call lizards ‘the new amphibians’ because of their elevated extinction risk (Gibbons et al. 2000; Sinervo et al. 2010). Climate change has contributed to these threats (Houlahan et al. 2000; Carey and Alexander 2003; Stuart et al. 2004), and correlative climate envelope models predict that climate change will cause extinction in 12–47% of endemic frogs and 11–49% of endemic reptiles (Thomas et al. 2004). Sinervo et al. (2010) predicted that 20% of lizard species will become extinct by 2080, assuming a limited potential for evolutionary responses. Overall, both amphibians and reptiles are declining, and more tenuous analyses link their declines to climate change. Climate change will likely decrease fitness substantially in some populations and thus could generate strong selection on climate-related traits. However, considerations of these threats rarely account for the mitigating effects of phenotypic plasticity and evolutionary adaptations.

Adaptive evolution and plasticity offer two ways species can sustain fitness despite climate change (Holt 1990; Urban et al. 2012). Biologists continue to debate the importance of these mechanisms in mediating climate change impacts (Gienapp et al. 2008; Hoffmann and Sgro 2011). Merilä and Hendry (2014) argue in this issue that neither adaptive evolution nor plasticity should be considered the null hypothesis for climate-related phenotypic change. We agree and further suggest that we should condition our predictions about their relative importance based on available empirical knowledge. Toward this end, we evaluate the degree to which amphibians and reptiles respond to climate variation through evolved genetic differences and environment-induced plasticity. We review studies that evaluate evolutionary or plastic response to spatial or temporal variation in climate and perform a meta-analysis on the proportion of traits showing climate-related differences. We also assess if those changes are adaptive and how accurately phenotypic differences map to climate change versus other correlated environmental variables.

### Adaptive evolution and plasticity in amphibians and reptiles

We now briefly outline the current understanding of evolution and plasticity with respect to climate responses in reptiles and amphibians before conducting our literature review and analysis. Plasticity occurs often in amphibians and reptiles – to such a degree that we expect plasticity in almost every trait we measure, ranging from behavior to morphology to life-history traits (Relyea 2001; Booth 2006; Urban 2010). Phenological shifts are especially common in amphibians (Beebee 1995; Gibbs and Briesch 2001; Todd et al. 2011), where changes in breeding phenology are two to four times stronger than responses in other taxonomic groups (Parmesan 2007). For instance, the dwarf salamander *Eurycea quadridigitata* migrates to breeding ponds 76 days later than it did 25 years ago (Todd et al. 2011). These phenological changes likely involve plasticity. However, the evolutionary contribution has never been directly measured, making it difficult for one to conclude about its importance. Lizards are expected to respond to climate change by altering sex ratios, habitat choice, and hatchling traits (Booth 2006). Scientists have argued both for (Gvozdik 2012) and against (Huey et al. 2009) the importance of plastic acclimation to warmer temperatures in buffering climate change effects in lizards. Huey et al. (2009) suggested that few tropical forest ectotherms demonstrate acclimation. In contrast, Gvozdik (2012) argued that plastic acclimation to higher temperatures might be common, but experimental approaches used to estimate plasticity often are conducted over extremely limited time spans or ignore additional sources of plasticity.

Can relatively long-lived amphibians and reptiles genetically adapt to climate change? One study parameterized a model of evolutionary responses to climate change based on estimates of phenotypic variation in critical thermal maxima and average evolutionary rates in amphibians and reptiles (Skelly et al. 2007). The authors predicted that critical thermal maxima could evolve 3.2°C in 50 years – fast enough to track climate changes. However, this estimate ignores potential limits to additive genetic variation at extreme temperatures or selection that is so strong that populations become extirpated before adaptation.

In contrast to amphibians, scientists have argued that reptiles are less likely to evolve temperature-related traits. This more pessimistic view stems from reptiles’ longer generation times relative to rapid climate change, low heritabilities, and genetic trade-offs among related traits (Janzen 1994; Schwanz and Janzen 2008; Sinervo et al. 2010). Sinervo et al. (2010) estimated that recently extirpated lizard populations had faced a standardized selection intensity of 0.34 on average. This strong selection intensity might be sustained under controlled conditions in a laboratory, but might often lead to extirpation in wild populations. However, empirical evidence remains limited in scope. For example, the evidence for low heritability in traits related to lizard persistence comes from a single population evaluated in the laboratory (Sinervo et al. 2010), and yet we know that heritabilities vary across species, populations, and environments. Also, genetic variation must be high enough to allow adaptation to rapid climate change. Genetic variation exists within and among populations for temperature-dependent sex determination in reptiles, but modeling studies suggest that it might not be sufficient to counter predicted levels of climate change (Morjan 2003; Mitchell and Janzen 2010). Hence, previous work suggests divergent views on the ability of amphibians and reptiles to adapt in response to climate change, setting the stage for a more synthetic analysis of the literature.

### Literature review and meta-analysis

To fill the gap in our understanding of evolutionary and plastic responses to climate variation, we searched Web of Science for articles using the following search terms: ‘amphibia*/reptile*’ AND (‘climate’ OR ‘global warming’) AND (‘plastic*’ OR ‘evol*’ OR ‘adapt*’ OR ‘selection’ OR ‘reaction norm’ OR ‘genotype by environment’ OR ‘GxE’ OR ‘phenotyp*’). We further searched literature citations of these articles for additional references. We read each article and only considered studies that presented empirical evidence for phenotypic or genotypic differences associated with climate change (e.g., temperature, precipitation). For each study, we recorded the climate change factor, phenotypic response (e.g., laying date, size, survival), and evaluated evidence for evolved or plastic differences for each species or population. We found 72 estimates recorded in 30 studies that estimated climate-related trait variation in 50 amphibian species and 29 estimates recorded in 24 studies conducted on 18 reptile species (Table 1).

**Table 1:** Summary of 26 studies on reptiles and 32 on amphibians designed to examine plastic and genetic responses of traits driven by climate variation

Common Name	Species	Trait Type	Factor	Genetic	Plastic	GxE	Adapt	Cause	Time	Reference
Reptiles										
Painted turtle	*Chrysemys picta*	Sex ratio	T	.	Y(4)	.	.	Y(2)	FD,MD	Janzen (1994)
American alligator	*Alligator mississipiensis*	Sex ratio	T	Y(2)	.	Y(2)	.	Y(1)	.	Rhen and Lang (1998)
Snapping turtle	*Chelydra serpentina*	Sex ratio		Y(2)	.	Y(2)	.	Y(1)	.	
Painted turtle	*Chrysemys picta*	Sex ratio		Y(2)	.	N(2)	.	Y(1)	.	
Snapping turtle	*Chelydra serpentina*	Nest site choice	L	.	Y(6)	.	.	Y(1,2)	.	Ewert et al. (2005)
		Nesting date		.	Y(6)	.	.	Y(1,2)	.	
		Incubation period	L & IT	Y(2,5)	.	.	.	Y(1)	.	
		Pivotal temperature*		Y(2,5)	.	.	Y(5)	Y(1)	.	
		Male-biased midrange*		N(2,5)	N(2,6)	.	.	.	.	
		Male-biased zone*		Y(2,5)	.	.	Y(5)	Y(1)	.	
Red-eared slider	*Trachemys scripta elegans*	Offspring size	T	.	N(4)	.	.	.	FD	Willette et al. (2005)
		Offspring size		.	N(4)	.	N(5)	.	FD	
		Energy stores		.	Y(4)	.	N(5)	Y(2)	FD	
Common lizard	*Lacerta vivipara*	Juvenile size	T	.	Y(4)	.	Y(5)	Y(2)	FD	Chamaille- Jammes et al. (2006)
		Adult size		.	Y(4)	.	Y(5)	Y(2)	FD	
Australia water dragon	*Physignathus lesueurii*	Nest site choice	L & E	.	Y(4)	.	Y(5)	Y(1)	.	Doody et al. (2006)
		Pivotal temperature*		.	N(3)	.	.	.	.	
Loggerhead	*Caretta caretta*	Nesting date	SST	.	Y(4)	.	.	Y(2)	FD	Hawkes et al. (2007)
Common lizard	*Lacerta vivipara*	Juvenile dispersal	T	.	Y(4)	.	.	Y(2)	FD	Massot et al. (2008)
Painted turtle	*Chrysemys picta*	Nesting date	T	.	Y(4)	.	.	Y(2)	FD	Schwanz and Janzen (2008)
Australia water dragon	*Physignathus lesueurii*	Nest depth	E	.	Y(6)	.	Y(5)	Y(1,2)	.	Doody (2009)
Three-lined skink	*Bassiana duperreyi*	Nest site choice	T	.	N(4)	.	N(5)	.	FD	Telemeco et al. (2009)
		Nest depth		.	Y(4)	.	Y(5)	Y(2)	FD	
		Nesting date		.	Y(4)	.	.	Y(2)	FD	
Spotted skink	*Niveoscincus ocellatus*	Sex ratio	T	.	Y(4)	.	.	Y(2)	FD	Wapstra et al. (2009)
Leatherback turtle	*Dermochelys coriacea*	Offspring size	NT	.	Y(4)	.	.	Y(1)	.	Mickelson and Downie (2010)
		Locomotor ability		.	Y(4)	.	.	Y(1)	.	
Black ratsnake	*Elaphe obsoleta*	Activity	L	.	N(5,6)	.	.	.	.	Sperry et al. (2010)
		Survival		.	N(5,6)	.	.	.	.	
		Overwinter survival		.	Y(5,6)	.	Y(5)	Y(1,2)	.	
Lizard	*Eremias multiocellata*	Incubation period	GT	.	Y(2,3)	.	.	Y(1)	.	Zhang et al. (2010)
		Sex ratio		.	Y(2,3)	.	.	Y(1)	.	
		Clutch size		.	Y(2,3)		.	Y(1)	.	
		Offspring size		.	N(2,3)	.	.	Y(1)	.	
Spotted skink	*Niveoscincus ocellatus*	Offspring date of birth	CD	.	Y(4)	.	.	Y(2)	FD	Cadby et al. (2011)
		Offspring size		.	N(4)	.	N(5)	.	FD	
Mary River turtle	*Elusor macrurus*	Incubation period	IT	.	Y(2,3)	.	.	Y(1)	.	Micheli- Campbell et al. (2011)
		Offspring size		.	Y(2,3)	.	.	Y(1)	.	
		Growth rate		.	Y(2,3)	.	.	Y(1)	.	
		Righting response		.	Y(2,3)	.	N(5)	Y(1)	.	
		Swimming performance		.	Y(2,3)	.	N(5)	Y(1)	.	
Large Psammodromus	*Psammodromus algirus*	Hatching success	IT	.	N(2,3)	.	.	.	.	Monasterio et al. (2011)
		Incubation period		.	Y(2,3)	.	.	Y(1)	.	
		Hatching date		.	Y(2,3)	.	.	Y(1)	.	
		Size		.	Y(2,3)	.	.	Y(1)	.	
		Body condition		.	Y(2,3)	.	Y(5)	Y(1)	.	
		Growth rate		.	Y(2,3)	.	Y(5)	Y(1)	.	
Rock lizard	*Iberolacerta cyreni*	Hatching success	IT	.	Y(2,3)	.	N(5)	Y(1)	.	
		Hatching date		.	Y(2,3)	.	.	Y(1)	.	
		Incubation period		.	Y(2,3)	.	.	Y(1)	.	
		Survival		.	Y(2,3)	.	N(5)	Y(1)	.	
		Size		.	Y(2,3)	.	.	Y(1)	.	
		Body condition		.	Y(2,3)	.	Y(5)	Y(1)	.	
		Growth rate		.	Y(2,3)	.	Y(5)	Y(1)	.	
Painted turtle	*Chrysemys picta*	Developmental rate	IT & TV	.	Y(2,3)	.	.	Y(1)	.	Neuwald and Valenzuela (2011)
		Embryonic mortality		.	Y(2,3)	.	N(5)	Y(1)	.	
		Sex ratio		.	Y(2,3)	.	.	Y(1)	.	
Sand lizard	*Lacerta agilis*	Sexual selection	T	.	N(4,5)	.	N(5)	.	FD	Olsson et al. (2011)
		Mate encounter rate		.	Y(4,5)	.	.	Y(2)	FD	
		Polyandry vs polygyny		.	Y(4,5)	.	.	Y(2)	FD	
		Sires per clutch		.	Y(4,5)	.	.	Y(2)	FD	
Three-lined skink	*Bassiana duperreyi*	Learning ability	IT	.	Y(2,3)	.	Y(5)	Y(1)	.	Amiel and Shine (2012)
Common lizard	*Zootoca vivipara*	Juvenile growth rate	CD (T&P)	.	Y(5)	.	.	Y(2)	FD	Le Galliard et al. (2010)
		Juvenile size		.	N(5)	.	.	.	FD	
		Subadult growth rate		.	Y(5)	.	.	Y(2)	FD	
		Subadult size		.	Y(5)	.	.	Y(2)	FD	
		Adult size		.	N(5)	.	.	.	FD	
		Adult growth rate		.	N(5)	.	.	.	FD	
		Juvenile survival		.	Y(4)	.	Y(5)	Y(2)	FD	
		Parturition date		.	Y(4)	.	.	Y(2)	FD	
Mary River turtle	*Elusor macrurus*	Survival	IT & TV	.	Y(2,3)	.	N(5)	Y(1)	.	Micheli- Campbell et al. (2012)
Painted turtle	*Chrysemys picta*	Nest site choice	NH	N(2,5)	Y(2,6)	.	.	Y(1,2)	.	Refsnider and Janzen (2012)
		Nest depth		N(2,5)	Y(2,6)	.	Y(5)	Y(1,2)	.	
		Nesting date		Y(2,5)	Y(2,6)	.	.	.	.	
Lizard	*Eremias multiocellata*	Incubation period		.	Y(2,3)	.	.	Y(1)	.	Tang et al. (2012)
		Sex ratio		.	Y(2,3)	.	.	Y(1)	.	
		Clutch size		.	N(2,3)	.	.	.	.	
		Offspring size		.	Y(2,3)	.	.	Y(1)	.	
		Female size		.	Y(2,3)	.	.	Y(1)	.	
Keelback snake	*Tropidonophis mairii*	Locomotor ability	IT	.	Y(2,3)	.	Y(5)	Y(1)	.	Bell et al. (2013)
Amphibians										
Spotted salamander	*Ambystoma maculatum*	Embryonic duration	T	.	Y(2,3)	.	.	Y(1)	.	Voss (1993)
		Body size at hatching		.	Y(2,3)	.	.	Y(1)	.	
		Stage at hatching		.	Y(2,3)	.	N(5)	Y(1)	.	
Natterjack toad	*Bufo calamita*	Spawning date	T	.	Y(4)	.	Y(5)	Y(1,2)	FD	Beebee (1995)
Common frog	*Rana temporaria*	Spawning date		.	Y(4)	.	Y(5)	Y(1,2)	FD	
Edible frog	*Rana kl. esculenta*	Spawning date		.	Y(4)	.	Y(5)	Y(1,2)	FD	
Smooth newt	*Triturus vulgaris*	Spawning date		.	Y(4)	.	Y(5)	Y(1,2)	FD	
Great crested newt	*Triturus cristatus*	Spawning date		.	Y(4)	.	Y(5)	Y(1,2)	FD	
Palmate newt	*Triturus helveticus*	Spawning date		.	Y(4)	.	Y(5)	Y(1,2)	FD	
Wood frog	*Rana sylvatica*	Embryonic hatching rate	T	N(2,5)	Y(2,3)	Y(2,5)	Y(5)	Y(1,2)	.	Skelly and Freidenburg (2000)
		Critical thermal maximum		Y(2,5)	.	.	Y(5)	Y(1,2)	.	
Western toad	*Bufo boreas*	Breeding date	T	.	N(4)	.	.	.	FD	Blaustein et al. (2001)
Western toad	*Bufo boreas*	Breeding date		.	Y(4)	.	Y(5)	Y(1,2)	FD	
Western toad	*Bufo boreas*	Breeding date		.	Y(4)	.	Y(5)	Y(1,2)	FD	
Cascades frog	*Rana cascadae*	Breeding date		.	N(4)	.	.	.	FD	
Cascades frog	*Rana cascadae*	Breeding date		.	Y(4)	.	Y(5)	Y(1,2)	FD	
Fowler's Toad	*Bufo fowleri*	Breeding date		.	N(4)	.	.	.	FD	
Spring peeper	*Pseudacris crucifer*	Breeding date		.	Y(4)	.	Y(5)	Y(1,2)	FD	
Wood frog	*Rana sylvatica*	Thermal preference	T	Y(2,5)	.	.	Y(5)	Y(1,2)	.	Freidenburg and Skelly (2004)
Italian agile frog	*Rana latastei*	Larval mass	T	Y(2,5)	.	.	.	.	.	Ficetola and Bernardi (2005)
		Time to metamorphosis		Y(2,5)	.	.	Y(5)	Y(1)	.	
		Mass at metamorphosis		N(2,5)	.	.	.	.	.	
Common midwife toad	*Alytes obstetricans*	Survival to metamorphosis	P&T (HY)	.	N(2,3)	.	.	.	.	Richter-Boix et al. (2006)
		Time to metamorphosis		.	Y(2,3)	.	Y(5)	Y(1)	.	
		Mass at metamorphosis		.	Y(2,3)	.	Y(5)	Y(1)	.	
Common parsley frog	*Pelodytes punctatus*	Survival to metamorphosis		.	N(2,3)	.	.	.	.	
		Time to metamorphosis		.	Y(2,3)	.	Y(5)	Y(1)	.	
		Mass at metamorphosis		.	Y(2,3)	.	Y(5)	Y(1)	.	
Common toad	*Bufo bufo*	Survival to metamorphosis		.	N(2,3)	.	.	.	.	
		Time to metamorphosis		.	N(2,3)	.	.	.	.	
		Mass at metamorphosis		.	Y(2,3)	.	Y(5)	Y(1)	.	
Natterjack toad	*Bufo calamita*	Survival to metamorphosis		.	N(2,3)	.	.	.	.	
		Time to metamorphosis		.	N(2,3)	.	.	.	.	
		Mass at metamorphosis		.	N(2,3)	.	.	.	.	
Mediterranean tree frog	*Hyla meridionalis*	Survival to metamorphosis	P&T (HY)	.	N(2,3)	.	.	.	.	Richter-Boix et al. (2006)
		Time to metamorphosis		.	Y(2,3)	.	Y(5)	Y(1)	.	
		Mass at metamorphosis		.	Y(2,3)	.	Y(5)	Y(1)	.	
Perez's frog	*Rana perezi*	Survival to metamorphosis		.	Y(2,3)	.	Y(5)	Y(1)	.	
		Time to metamorphosis		.	Y(2,3)	.	Y(5)	Y(1)	.	
		Mass at metamorphosis		.	N(2,3)	.	.	.	.	
Pacific treefrog	*Pseudacris regilla*	Survival	T	.	Y(4,5)	.	Y(5)	Y(1,2)	FD	Govindarajulu and Anholt (2006)
Marsh frog	*Rana ridibunda*	Body length	P&T	.	Y(4)	.	.	Y(1,2)	FD	Tryjanowski et al. (2006)
Pool frog	*Rana lessonae*	Body length		.	Y(4)	.	.	Y(1,2)	FD	
Edible frog	*Rana esculenta*	Body length		.	Y(4)	.	.	Y(1,2)	FD	
Red back salamander	*Plethodon cinereus*	Color phenotype	T	Y†	.	.	Y(5)	Y(1)	FD	Gibbs and Karraker (2006)
Italian newt	*Triturus italicus*	Time to hatching	T	.	Y(2)	.	Y(5)	Y(1)		D'Amen et al. (2007)
Italian crested newt	*Triturus carnifex*	Time to hatching		.	Y(2)	.	Y(5)	Y(1)		
Common frog	*Rana temporaria*	Spawning date	T	.	Y(4)	.	Y(5)	Y(1,2)	FD	Sparks et al. (2007)
		Spawning date		.	Y(4)	.	Y(5)	Y(1,2)	FD	
		Spawning date		.	Y(4)	.	Y(5)	Y(1,2)	FD	
Common toad	*Bufo bufo*	Spawning date		.	Y(4)	.	Y(5)	Y(1,2)	FD	
		Migration to ponds		.	Y(4)	.	Y(5)	Y(1,2)	FD	
Common toad	*Bufo bufo*	Body condition	T	.	Y(4)	.	N(5)	Y(1)	FD	Reading (2007)
		Survival		.	Y(4)	.	N(5)	Y(1)	FD	
Tokyo salamander	*Hynobius tokyoensis*	Spawning date	T	.	Y(4)	.	Y(5)	Y(1,2)	FD	Kusano and Inoue (2008)
Montane brown frog	*Rana ornativentris*	Spawning date		.	Y(4)	.	Y(5)	Y(1,2)	FD	
Forest green treefrog	*Rhacophorus arboreus*	Spawning date		.	Y(4)	.	Y(5)	Y(1,2)	FD	
Palmate newt	*Lissotriton helveticus*	Number of eggs	T	.	Y(2,3)	.	N(5)	Y(1)	.	Galloy and Denoel (2010)
		Oviposition period		.	Y(2,3)	.	.	Y(1)	.	
		Hatching success		.	Y(2,3)	.	N(5)	Y(1)	.	
		Time to hatching		.	Y(2,3)	.	.	Y(1)	.	
		Oviposition rate		.	N(2,3)	.	.	.	.	
		Hatching rate		.	N(2,3)	.	.	.	.	
Common frog	*Rana temporaria*	Spawning date	T	Y(5)	Y	.	Y(4,5)	Y(1,2)	FD	Phillimore et al. (2010)
Common frog	*Rana temporaria*	Melanism	T (L)	.	Y(6)	.	Y(5)	Y(1,2)	.	Alho et al. (2010)
		Melanism	T	N(2,5)	Y(2,3)	N(2,5)	.	Y(1)	.	
Hourglass treefrog	*Dendropsophus ebraccatus*	Stage at hatching	P (HD)	.	Y(2,3)	.	N(5);Y(5)	Y(1)	.	Touchon and Warkentin (2010)
		Larval mortality		.	Y(2,3)	.	N(5);Y(5)	Y(1)	.	
		Predation vulnerability		.	Y(2,3)	.	Y(5)	Y(1)	.	
		Size at metamorphosis		.	Y(2,3)	.	.	Y(1)	.	
Pool frog	*Rana lessonae*	Survival	T	N(2,5)	N(2,3)	N(2,5)	.	.	.	Orizaola et al. (2010)
		Time to metamorphosis		Y(2,5)	Y(2,3)	Y(2,5)	Y(5)	Y(1)	.	
		Mass at metamorphosis		N(2,5)	Y(2,3)	N(2,5)	.	Y(1)	.	
		Size at metamorphosis		N(2,5)	Y(2,3)	N(2,5)	.	Y(1)	.	
		Larval growth rate		Y(2,5)	Y(2,3)	Y(2,5)	.	Y(1)	.	
Bullfrog	*Rana catesbeiana*	Body size	T (E)	.	Y(6)	.	Y(5)	Y(1,2)	.	Liu et al. (2010)
Panamanian golden frog	*Atelopus zeteki*	Chytrid Bd infection	T	.	Y(6)	.	Y(5)	Y(1,2)	FD	Richards- Zawacki (2010)
Hokkaido salamander	*Hynobius retardatus*	Body size	T	.	Y(2)	.	.	Y(1)	.	Michimae (2011)
		Time to metamorphosis		.	Y(2)	.	Y(5)	Y(1)	.	
Common frog	*Rana temporaria*	Leg length	T (L)	Y(2,5)	Y(2)	.	.	Y(1,2)	.	(Alho et al. 2011)
Bullfrog	*Rana catesbeiana*	Chytrid Bd infection	T	.	Y(2,3)	.	Y(5)	Y(1,2)	.	Chatfield and Richards- Zawacki (2011)
Northern cricket frog	*Acris crepitans*	Chytrid Bd infection		.	Y(2,3)	.	Y(5)	Y(1,2)	.	
Lowland leopard frog	*Rana yavapaiensis*	Chytrid Bd infection	T	.	Y(6)	.	Y(5)	Y(1,2)	.	Forrest and Schlaepfer (2011)
Dwarf salamander	*Eurycea quadridigitata*	Median arrival date	T & P	.	Y(4)	.	Y(5)	Y(1,2)	FD	Todd et al. (2011)
Marbled salamander	*Ambystoma opacum*	Median arrival date		.	Y(4)	.	Y(5)	Y(1,2)	FD	
Tiger salamander	*Ambystoma tigrinum*	Median arrival date		.	Y(4)	.	Y(5)	Y(1,2)	FD	
Ornate chorus frog	*Pseudacris ornata*	Median arrival date		.	Y(4)	.	Y(5)	Y(1,2)	FD	
Mole salamander	*Ambystoma talpoideum*	Median arrival date		.	N(4)	.	.	.	FD	
Southern toad	*Bufo terrestris*	Median arrival date		.	N(4)	.	.	.	FD	
Eastern narrowmouth toad	*Gastrophryne carolinensis*	Median arrival date		.	N(4)	.	.	.	FD	
Spring peeper	*Pseudacris crucifer*	Median arrival date		.	N(4)	.	.	.	FD	
Southern leopard frog	*Rana sphenocephala*	Median arrival date		.	N(4)	.	.	.	FD	
Eastern spadefoot toad	*Scaphiopus holbrookii*	Median arrival date		.	N(4)	.	.	.	FD	
Wood frog	*Rana sylvatica*	Peak calling date	T	.	Y(4)	.	Y(5)	Y(1,2)	FD	Walpole et al. (2012)
Spring peeper	*Pseudacris crucifer*	Peak calling date		.	Y(4)	.	Y(5)	Y(1,2)	FD	
Northern leopard frog	*Rana pipiens*	Peak calling date		.	Y(4)	.	Y(5)	Y(1,2)	FD	
Green frog	*Rana clamitans*	Peak calling date		.	N(4)	.	.	.	FD	
Bullfrog	*Rana cateseiana*	Peak calling date		.	N(4)	.	.	.	FD	
American toad	*Bufo americanus*	Peak calling date		.	N(4)	.	.	.	FD	
Boreal chorus frog	*Pseudacris maculata*	Mortality	P&T (HY)	N(2,5)	N(2,3)	N(2,5)	.	.	.	Amburgey et al. (2012)
		Time to metamorphosis		Y(2,5)	N(2,3)	N(2,5)	.	Y(1)	.	
		Size at metamorphosis		Y(2,5)	N(2,3)	N(2,5)	.	Y(1)	.	
Natterjack toad	*Bufo calamita*	Breeding habitat selection	T (L)	.	Y(6)	.	Y(5)	Y(1,2)	.	Rannap et al. (2012)
Gunther's toadlet	*Pseudophryne guentheri*	Embryonic survival	P (SM)	Y(2)	Y(2)	Y(2,5)	N(5)	Y(1)	.	Eads et al. (2012)
		Time to hatching		Y(2)	Y(2)	Y(2,5)	N(5)	Y(1)	.	
		Body size		Y(2)	Y(2)	Y(2,5)	N(5)	Y(1)	.	
Striped marsh frog	*Limnodynastes peronii*	Age at hatching	T	.	Y(2,3)	.	Y(5)	Y(1)	.	Niehaus et al. (2012)
		Size at hatching		.	Y(2,3)	.	.	Y(1)	.	
		Larval growth rate		.	Y(2,3)	.	Y(5)	Y(1)	.	
		Larval developmental rate		.	Y(2,3)	.	Y(5)	Y(1)	.	
		Age at hatching	TV	.	N(2,3)	.	.	.	.	
		Size at metamorphosis		.	Y(2,3)	.	.	Y(1)	.	
		Mass at metamorphosis		.	Y(2,3)	.	.	Y(1)	.	
Common frog	*Rana temporaria*	Activity level	T (L)	Y(2,5)	Y(2)	Y(2,5)	Y(5)	Y(1,2)	.	Orizaola et al. (2013)
		Body morphology		Y(2,5)	Y(2)	N(2,5)	.	Y(1,2)	.	
		Larval period		Y(2,5)	Y(2)	N(2,5)	Y(5)	Y(1,2)	.	
		Mass at metamorphosis		Y(2,5)	Y(2)	N(2,5)	.	Y(1,2)	.	
		Growth rate		Y(2,5)	Y(2)	N(2,5)	Y(5)	Y(1,2)	.	

A ‘Y’ indicates that evidence was found for genetic or plastic responses in traits or that adaptability or causality was investigated; ‘N’ indicates evidence was not found; ‘.’ indicates that it was not investigated. Numbers next to a ‘Y’ or ‘N’ denote the method of investigation invoked. **FACTOR** (climate factor proxy): CD – climate data, E – elevation, GT – gestation temperature, HD – hydration, HY – hydroperiod, IT – incubation temperature, L – latitude, NH – nest habitat, NT – nest temperature, P – precipitation, SM – soil moisture, SST – sea surface temperature, T – temperature, TV – temperature variability; **GENETIC** categories: 1 – animal models, 2 – common garden studies, 3 – comparison to model predictions, 4 – experimental evolution, 5 – space for time substitution, 6 – molecular genetic approaches; **PLASTIC** categories: 1 – animal models, 2 – common garden studies, 3 – experimental studies, 4 – fine-grained population responses, 5 – individual plasticity in nature, 6 – space for time substitution; **GxE** categories: 1 – animal models, 2 – common garden studies, 3 – comparison to model predictions, 4 – experimental evolution, 5 – space for time substitution; **ADAPT** categories: 1 – reciprocal transplants, 2 – phenotypic selection estimates, 3 – genotypic selection estimates, 4 – *Q*_st-_*F*_st_ comparison, 5 – common sense; **CAUSE** categories: 1 – common sense, 2 – phenotype by environment interactions, 3 – experimental selection/evolution; for full descriptions of all categories see Merilä and Hendry (this volume); **TIME** (time component included in data collection): RS – resurrection study, EX – field or greenhouse experiment through time, FD – field observations through time, MD – modeled through time.

* Feature of temperature-dependent sex determination.

† Other studies show color phenotype has a genetic basis.

We considered studies that evaluated phenotypic or genotypic differences in response to temporal (allochronic studies) and spatial variation (synchronic studies) in climate. Allochronic studies evaluate phenotypic responses to climate change over time and provide the strongest inferences (Hendry and Kinnison 1999; Merilä and Hendry 2014). The alternative to the allochronic study is the synchronic one, which documents phenotypic variation in space and requires that space can be substituted for time. The space-for-time substitution offers weaker inferences especially for genetic change. For instance, a population that adapted to climate variation in the past might not adapt to future climate change if genetic variation is lacking. Substituting space for time likely provides more robust predictions for phenotypic plasticity; plasticity that is induced by spatial climate variation is also likely to be induced by climate variation through time.

We only uncovered one study of allochronic genetic change in response to climate change in amphibians or reptiles and this one did so indirectly (Phillimore et al. 2010). Another study evaluated genetic studies through time, but not necessarily on the same populations (Gibbs and Karraker 2006). Two other studies evaluated spatial differences among populations that had experienced divergence in temperature over time and thus represents a more robust case for applying a space-for-time substitution (Skelly and Freidenburg 2000; Freidenburg and Skelly 2004). Sixty-three percent of amphibian estimates and 38% of reptile estimates were based on variation across space rather than time. To understand if these disparate designs affected overall outcomes, we tested for an effect of study design.

We performed a meta-analysis on the proportion of traits that contributed significantly to genetic adaptation, plasticity, and genotype-by-environment interactions. We logit-transformed proportions and weighted each study by the inverse expected variance or *np*/(1–*p*) where *n* is the number of traits analyzed in each study and *p* is the proportion of traits showing a significant difference (Lipsey and Wilson 2000). We added 0.5 to studies with zero proportions in order to include them (Veichtbauer 2010). We counted the responses of different species and different populations reported in the same study as separate estimates. However, we included study, species, and populations as random effects to control for their nonindependence in analyses.

We conducted analyses in R (v. 3.0.0) and used the lmer function to conduct mixed-effects models. The significance of random effects was assessed using a likelihood ratio test (Pinheiro and Bates 2000). We also tested for effects of study design (allochronic versus synchronic) and used the pvals function in R to calculate significance values.

### Traits

We used a word cloud algorithm (http://www.wordle.net) to visualize the frequency with which researchers measured different traits. This algorithm sets word frequencies proportional to font sizes. We arranged traits alphabetically from left to right. We included all traits analyzed across all studies while also consolidating traits into more general categories (e.g., ‘breeding date’ represents peak calling date, first date of arrival, and observation of egg deposition).

**Figure 1:**
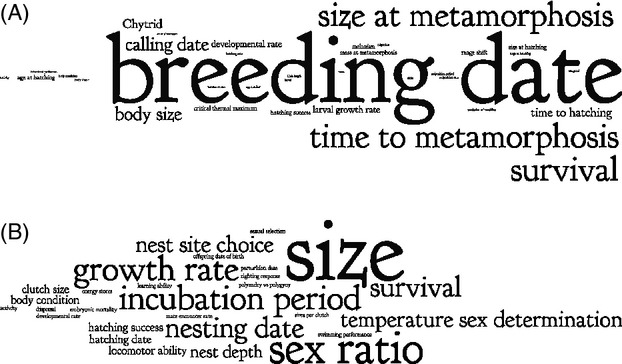
Word clouds indicating most commonly analyzed climate-change-related traits for (A) amphibians and (B) reptiles. Traits are listed alphabetically from left to right with font size set to the number of times the trait was studied relative to others.

Most studies on amphibians focused on phenological changes in breeding (Table 1; Fig. 1A; 31% of traits analyzed). Research also focused on amphibian life-history traits related to larval growth (14%), survival (9%), and embryonic development (8%). We found fewer measures of direct physiological effects of temperature (Skelly and Freidenburg 2000), resistance to climate-related diseases (Chatfield and Richards-Zawacki 2011; Forrest and Schlaepfer 2011), or adult traits other than breeding phenology (Tryjanowski et al. 2006; Reading 2007; Alho et al. 2010), despite the potential importance of these traits in mediating climate threats (Carey and Alexander 2003; Corn 2005; Pounds et al. 2006). Reptile studies focused on a wider range of traits including adult and offspring size (19%), sex determination threshold and sex ratios (15%), nesting site choice and depth (9%), incubation period (8%), and survival (8%; Fig. 1B).

### Evidence for plastic responses to climate variation

**Figure 2:**
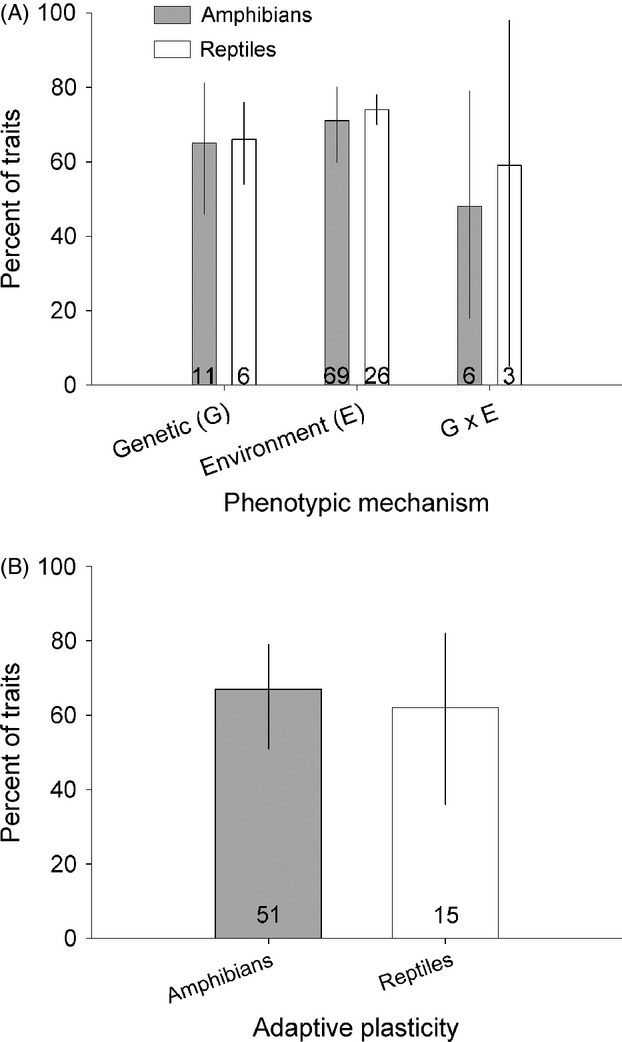
(A) Percent of traits from weighted proportional meta-analysis (±95% confidence intervals) that show significant genetic, environment, and genotype-by-environment contributions to responses to climate change and (B) percent of studies recording adaptive plasticity for amphibians (gray) and reptiles (white). Individual species and populations within a given study are treated as separate estimates. Numbers within bars denote the total number of examples that analyzed a given phenotypic mechanism.

The inverse-variance weighted proportion of plastic responses to climate variation in amphibians was 0.71 (95% confidence interval: 0.60, 0.80; Fig. 2A) based on 69 estimates. The random effects of species, study, and population did not significantly explain additional variation. The unweighted model indicated a similar proportion (= 0.70). Study design (allochronic versus synchronic) did not significantly alter the estimated proportion of plastic responses to climate variation (*P* = 0.220).

The inverse-variance weighted proportion of plastic responses to climate variation in reptiles was 0.69 (0.53, 0.81; Fig. 2A), based on 26 estimates. We detected a significant random effect of species (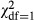 = 6.2, *P* = 0.012) but not of study or population. The unweighted model indicated a similar proportion (= 0.70). Whether the study design was allochronic or synchronic did not significantly alter the estimated proportion of plastic responses to climate variation (*P* = 0.722).

Many of the collected studies evaluated how amphibians and reptiles adjusted their breeding phenology in response to warmer climates (Beebee 1995; Blaustein et al. 2001; Schwanz and Janzen 2008; Telemeco et al. 2009; Todd et al. 2011). In general, the phenological responses of amphibian breeding are among the largest observed – estimated to be two to four times stronger than those in other taxonomic groups (Parmesan 2007). Thus, the plastic response to climate change is well established and strong in amphibians. Generally, these rapid changes in phenology are assumed to occur too quickly to entail evolution. However, the genetic component of this response has never been evaluated in amphibians or reptiles in common garden experiments. One study estimated no additive genetic variance for breeding phenology in painted turtles, but moderate genetic variation emerged following a warm winter (*V*_*A*_ = 0.13; evolvability CV_*A*_ of 0.23; McGaugh et al. 2010). Another study evaluated variation in temperature-dependent breeding dates along a latitudinal gradient and used observed differences in reaction norms to argue for the evolution of breeding phenology (Phillimore et al. 2010). Although the differences in reaction norms are compelling, other uncontrolled environmental factors could explain these differences. Applying complicated statistics to observational data cannot replace the simple elegance of the common garden experiment. Some of these phenological changes over longer time periods might involve genetic changes, but we lack clear evidence to date. Introducing a population from another climate region into a common environment and evaluating differences in phenology between introduced and native populations under the same climate cues would indicate if these responses diverge genetically across landscapes. Determining if they evolve in response to climate change is more difficult, requiring researchers to identify candidate genes, parameterize genetic models, or experimentally estimate trait changes through time.

Temperature-dependent sex determination presents another important case of plasticity with strong links to climate (Janzen 1994; Mitchell and Janzen 2010). Warmer climates generally bias sex ratios in reptiles toward females. Some populations could face demographic collapse if high temperatures preclude the development of one sex (Janzen 1994). Yet, multiple studies suggest that reptiles can respond behaviorally through nesting site choice, depth, and breeding time (Doody et al. 2006; Doody 2009; Refsnider and Janzen 2012). These behaviors could mediate the effect of hotter temperatures on sex ratios. For instance, water dragons (*Physignathus lesueurii*) maintain similar incubation temperatures despite changing environmental temperatures by digging shallower nests and selecting more open nest sites in colder regions (Doody et al. 2006; Doody 2009). In contrast, changes in breeding time and nest depth did not buffer an observed 1.6°C warming period for the lizard *Bassiana duperreyi*. These lizards nested 4 weeks earlier and buried eggs 15 mm deeper, but eggs still warmed 1.5°C, which threatened their normal sex ratios (Telemeco et al. 2009). Behaviors might moderate changes in sex ratios, but not always to the degree necessary to mitigate climate change.

Different thermal regimes were associated with plastic changes in life-history traits such as clutch size, development rate, growth rate, and survival in amphibians (Voss 1993; Galloy and Denoel 2010; Orizaola et al. 2010; Niehaus et al. 2011). Some of these changes were dramatic. For instance, half as many eggs were laid by palmate newts (*Lissotriton helveticus*) when raised at 22 vs 18°C (Galloy and Denoel 2010). Experimentally induced shorter hydroperiods altered survival, development, and growth in multiple European frogs and toads (Richter-Boix et al. 2006). In contrast, simulated drying did not induce plastic changes in boreal chorus frogs (*Pseudacris maculata*; Amburgey et al. 2012).

Results suggest that plasticity will generally buffer some of the effects of climate change. However, not all traits are plastic, and sometimes plasticity might prove insufficient by itself to buffer climate change.

### Evidence for genetic responses to climate variation

Our sample size was small and only one study explicitly evaluated genetic changes over time in response to climate change. However, all 17 studies that evaluated genetic differences found support for adaptive genetic differences in at least one trait. The estimated weighted proportion of genetic responses to climate variation in amphibians was 0.65 (95% confidence interval: 0.46, 0.81), and the proportion in reptiles was 0.66 (0.43, 0.83; Fig. 2A). The unweighted and weighted estimates did not differ substantially from each other, and random effects were not significant for amphibians and reptiles.

In one amphibian example of a genetic response to climate variation, wood frogs (*Rana sylvatica*) evolved different critical thermal maxima and thermal preferences between warm and cold ponds that had diverged in temperature over 36 years (Skelly and Freidenburg 2000; Freidenburg and Skelly 2004). In another case, warmer climates led to increasing proportions of dark morphed red-backed salamanders (*Plethodon cinereus*; Gibbs and Karraker 2006). Pool frog tadpoles (*Rana lessonae*) diverged in temperature-dependent developmental and growth rates across habitats that differed in temperature (Orizaola et al. 2010). For reptiles, snapping turtles (*Chelydra serpentina*) from different latitudes differed in their temperature-dependent sex determination patterns when raised in the laboratory (Ewert et al. 2005), and sex ratios differed among families for alligators and two turtle species (Rhen and Lang 1998). The overall magnitude of these changes was often not very large. For instance, the study on the evolution of critical thermal maxima in wood frog tadpoles found a difference of 0.4°C between populations inhabiting warm and those inhabiting cold ponds (Skelly and Freidenburg 2000). Similarly, the occurrence of striped red-backed salamander morphotypes decreased by only 6% over the last century (Gibbs and Karraker 2006).

We only found six amphibian and three reptile estimates of genotype-by-environment interactions in response to climate variation. The reptile study (Rhen and Lang 1998) found evidence for genotype-by-environment interactions in two of the three species they examined. For amphibians, we had enough studies to apply a weighted meta-analysis. The estimated proportion of genotype-by-environment responses to climate variation was 0.48 (95% confidence interval: 0.13, 0.85; Fig. 2A). The unweighted estimate was similar (= 0.47), and random effects were not significant. One genotype-by-climate interaction occurred when *R. sylvatica* tadpoles varied in hatching depending both on if they came from warm or cold ponds and their exposure to cool or warm environmental conditions (Skelly and Freidenburg 2000). In *R. lessonae* tadpoles, larval growth rate and time to metamorphosis were accelerated the most in warm conditions for the population originating from the coldest environment, indicating countergradient variation (Conover and Schultz 1995; Orizaola et al. 2010).

Other research suggests the potential for genetic variation to fuel the evolution of sex determination thresholds in reptiles in response to warmer climates. A review of reptiles with temperature-dependent sex determination reports heritabilities ranging from 0.26 to 0.82 (McGaugh and Janzen 2011). Morjan (2003) parameterized a model to predict that threshold temperatures might evolve more quickly than nest site choice, but not rapidly enough to counter predicted climate change. Morjan (2003) finds even slower rates of evolution of nest site choice owing to low heritability and high maternal contributions. Heritability of nest site choice in painted turtles (*Chrysemys picta*) ranged from 0.06 to 0.7 (McGaugh et al. 2010). Additive genetic variance increased in this trait following warm winters (McGaugh et al. 2010), which demonstrates that extreme future climates might reveal additive genetic variation to fuel adaptation exactly when it is needed most. However, in other cases, limits to additive genetic variation might exist that would prevent evolution in response to more extreme climate variation. More generally, we highlight the many opportunities for researchers to perform novel experiments that test for the genetic basis of climate-related traits in amphibians and reptiles.

On a cautionary note, few studies controlled for transgenerational effects, which could affect responses to climate change. Transgenerational effects occur when nongenetic contributions originate from previous generations (e.g., maternal effects). In addition, all our evidence for evolution arose from genetic differences measured across populations rather than through time. This space-for-time substitution is criticized as much as it is applied, but persists because of limited data. The problem is that past adaptive divergence among populations need not indicate the potential for future adaptation, especially at the rates required by climate change. Spatial differences might have evolved over centuries, whereas climate change might require evolution over decades. Also, strong natural selection could produce demographic collapse before population-level fitness can recover (Gomulkiewicz and Holt 1995; Sinervo et al. 2010).

On a more positive note, the studies on *R. sylvatica* tadpoles revealed genetic differences between ponds that had undergone known temporal changes in temperature over just a few decades (Skelly and Freidenburg 2000; Freidenburg and Skelly 2004), suggesting that space can substitute for time when we have information on recent environmental changes across the landscape. At least some evolutionary rates might be sufficient to meet the demands of the changing climate.

### Are traits adaptive?

We next assessed if trait differentiation was adaptive or maladaptive based on authors’ conclusions and logic. In the latter case, we assumed a trait was maladaptive if it reduced fecundity or survival. Only a few studies examining genetic variation also provided information that could be used to assess if changes were adaptive. Of those studies, all six amphibian studies and the one reptile study indicated an adaptive origin for genetic changes.

Climate change factors generally induced adaptive plasticity (Fig. 2B). The weighted proportion of plastic responses to climate variation in amphibians that was considered adaptive was 0.67 (95% confidence interval: 0.51, 0.79). The random effect of species was not significant (*P* = 0.813), but study and population significantly explained additional variation (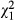 = 26.1, 8.2, respectively; *P* < 0.005). The unweighted model indicated the same proportion. The proportion of plastic responses to climate variation in reptiles that was considered adaptive was 0.62 (95% confidence intervals: 0.36, 0.82). The random effects of species, study, and population did not significantly explain additional variation. The unweighted model indicated the same proportion. For both amphibians and reptiles, allochronic studies did not show significantly different proportions of adaptive variation when compared against synchronic studies (*P* > 0.6).

Although most observed plasticity was assumed to be adaptive, we also detected substantial maladaptive plasticity (Fig. 2B). Hotter temperatures enhanced learning performance in the lizard *B. duperreyi* (Amiel and Shine 2012) and accelerated development and increased juvenile body condition in lacertid lizards (Monasterio et al. 2011). However, higher incubation temperatures produced lower hatching success, slower righting responses, poorer swim performance, and skewed sex ratios in other reptiles (Janzen 1994; Wapstra et al. 2009; Mickelson and Downie 2010; Micheli-Campbell et al. 2011; Bell et al. 2013). Despite the near-universality of plastic responses to climate variation, some plasticity will ultimately prove maladaptive under altered climates. Understanding the fitness consequences of climate-induced plasticity requires researchers to delineate plastic responses in experiments, create artificial phenotypes independent of genotype (Waddington 1953), and then track their long-term fitness in experimental or natural situations.

### Which selective force?

We also evaluated the degree to which studies evaluated the causal linkage between climate change and each phenotypic response. Although multiple methods exist (Merilä and Hendry 2014), our studies assessed causality using common sense, phenotype–environment correlations, or experimental manipulations. Climate was suggested to be involved in phenotypic changes in all the traits analyzed (Table 1). However, these conclusions were often based on common sense rather than rigorous tests.

## Future directions

Given limited information on adaptive responses to climate change, we argue that we need to intensify our efforts to explore how plasticity and genetic adaptation will attenuate the future impacts of climate change. These mitigating factors might not provide a panacea, but we should estimate their contributions and include them in any rigorous assessment of climate change effects on species. Most of the examples we found required substituting space for time and thus constituted weaker inferences. Quantitative genetic models might be used to predict future phenotypes, but they assume that we can accurately estimate the heritabilities of traits and strength of selection in the wild (Endler 1986), which is often not the case (Hoffmann and Merilä 1999; Charmantier and Garant 2005). Genetic correlations can constrain evolutionary responses to climate change (Etterson and Shaw 2001). Moreover, attempts to predict evolution in the wild have frequently failed. These shortcomings are due to (i) poor estimates of heritability or selection in the wild, (ii) the ability of selection to act on nonheritable phenotypic variation, (iii) the fact that traits are genetically correlated, (iv) rapidly shifting environments, or (v) because of a simple lack of statistical power (Merilä et al. 2001). All of these reasons cast doubt on our ability to extrapolate current evolutionary responses far into the future.

Other options include using analogous changes in landscapes as natural experiments (Skelly and Freidenburg 2000) or performing common garden experiments through time, where relevant phenotypic responses are evaluated in the same populations and using the same methods across long enough periods to capture climate change. These rigorous experiments would reveal evolution in real time, highlight if evolution is keeping up with changing climate, and provide insights into the limits of additive genetic variation to fuel evolutionary responses in the near future. Experimental evolution in the wild provides another compelling option. Researchers could design experiments that manipulate climate factors directly and evaluate evolutionary changes in focal populations or transplant populations into selection regimes that mimic future climates and then track their evolution. However, the lack of allochronic studies in amphibians and reptiles is clearly related to the fact that these species often have long generation times that prevent such comparisons being made over practical time spans. Lastly, a candidate genes approach offers many advantages if sufficient genomic and trait-gene mapping is available or can be accomplished.

## Conclusions

Many amphibians and reptiles have already undergone climate-related extinctions or declines (Gibbons et al. 2000; Parmesan and Yohe 2003; Pounds et al. 2006; Sinervo et al. 2010), and others are approaching the edge of their thermal tolerance (Huey et al. 2009). Models predict future declines in amphibians and reptiles owing to climate change (Thomas et al. 2004; Malcolm et al. 2006). However, these correlative models rarely account for evolutionary change. Strong plasticity or high genetic variance might prove sufficient to generate phenotypic changes that match the demands of a new climate for some species. Alternatively, relatively long generations and limited genetic variation could reduce their capacity for adaptive change after plastic reserves have been exhausted (Morjan 2003; Sinervo et al. 2010).

Here, we show that both adaptation and plasticity could play important roles in mitigating climate change for amphibians and reptiles, but data is still limited, especially for genetic responses. Our literature review suggests that plasticity might often mediate changes to climate in both taxonomic groups. A long history of research has focused on plasticity in these species, and we show here that changing climates might often induce adaptive plasticity. Although evidence for genetic adaptations to climate was limited to a handful of mostly synchronic studies, all of these studies found evidence for climate adaptation in at least one trait. Similar assessments of other taxonomic groups in this issue provide evidence for widespread plastic and adaptive responses to climate variation and concurrently highlight the same lack of data for evolutionary responses (Franks et al. 2014; Reusch 2014; Schilthuizen and Kellermann 2014). If future studies confirm these results, then plasticity and adaptive capacity could dampen some of the doom and gloom associated with research on biotic responses to climate change.
